# No Evidence for an Association of Vitamin D Deficiency and Migraine: A Systematic Review of the Literature

**DOI:** 10.1155/2014/827635

**Published:** 2014-05-08

**Authors:** Giuseppe Lippi, Gianfranco Cervellin, Camilla Mattiuzzi

**Affiliations:** ^1^Laboratory of Clinical Chemistry and Hematology, Academic Hospital of Parma, 43126 Parma, Italy; ^2^U.O. Diagnostica Ematochimica, Azienda Ospedaliero-Universitaria di Parma, 14 Via Gramsci, 43126 Parma, Italy; ^3^Emergency Department, Academic Hospital of Parma, 43126 Parma, Italy; ^4^Service of Clinical Governance, General Hospital of Trento, 38122 Trento, Italy

## Abstract

Vitamin D deficiency is associated with a number of human disorders, including cardiovascular disease, cancer, diabetes, frailty, and infections. Since an association between vitamin D and migraine has also been recently speculated, we performed an electronic search on Medline, Scopus, and Web of Science using the keywords “migraine” and “vitamin D,” “25OH-D” “cholecalciferol,” “ergocalciferol,” with no language or date restriction. The electronic search allowed identifying seven studies (3 observational, 2 cross-sectional, and 2 case reports). The two case reports, including four women, showed favourable effects of vitamin D supplementation on migraine severity, but these studies were small and not placebo controlled. As regards the three observational studies, vitamin D deficiency was observed in 13.2 to 14.8% of migraine patients, and these rates do not differ from those reported in the general population (i.e., vitamin D deficiency between 22 and 42%). The results of the two cross-sectional studies are even more controversial, since no association was found between vitamin D status and migraine in both trials. In conclusion, the current evidence suggests that the association between migraine and vitamin D lacks reliable scientific support.

## 1. Introduction

The generic term “vitamin D” comprehends a number of fat-soluble secosteroids that exert a multitude of biological functions in vertebrates. In humans, the most important compounds are represented by ergocalciferol (i.e., vitamin D2) and cholecalciferol (i.e., vitamin D3) [1]. The former molecule is not constitutively produced by plants or vertebrates but mainly originates from phytoplankton, fungi, and invertebrates. At variance with ergocalciferol, cholecalciferol is actively synthesized in humans and can also be assumed with the diet by ingestion of animal sources, especially liver, fish, eggs, or yolks [1].

The keratinocytes of the skin are the leading source of vitamin D in humans. In brief, previtamin D is synthesized nonenzymatically from 7-dehydrocholesterol during exposure to the UV rays and then undergoes a temperature-dependent rearrangement to generate cholecalciferol [2]. The synthesis of 25-hydroxyvitamin D (25OH-D) is typically dependent upon vitamin D hydroxylation in the liver, which is then followed by a further hydroxylation in the kidneys to generate 1-*α*,25-dihydroxycholecalciferol (i.e., calcitriol or 1,25OH-2D). According to this biologic pathway, the concentration of total vitamin D (i.e., 25OH-D) is currently regarded as the most suitable indicator of total vitamin D body stores [3].

Besides the well-established function on bone metabolism [4], low levels of vitamin D have been recently implicated in a number of human disorders, including cardiovascular disease [5], cancer [6], diabetes [7], frailty [8], and infections [9]. Interestingly, an association between vitamin D concentration and migraine has also been recently speculated by Prakash et al., who reported an increased frequency of headache attacks in autumn-winter and a reduced frequency in summer, a trend that closely mirrors the seasonal variations of serum vitamin D levels [10]. Migraine is a highly prevalent condition, which is currently ranked third among the most frequent human diseases and seventh among the leading causes of disability worldwide [11]. The pathogenesis of this condition is complex and multifaceted and basically involves an initial decrease of cerebral blood flow, which is then followed by reactive vasodilatation, plasma protein extravasation, and sterile inflammation in association with hypersensitization of central pain pathways [12]. Recent evidence also suggests that migraine with or without aura shares similar pathogenetic and clinical aspects, wherein the aura of migraine cannot be biologically disconnected from the actual headache attack [13].

As such, the aim of this paper is to provide an overview of the current scientific literature about the potential epidemiological association between serum vitamin D and migraine.

## 2. Search Criteria

We performed an electronic search on Medline, Scopus, and Web of Science using the keywords “migraine” and “vitamin D,” “25OH-D,” “cholecalciferol,” or “ergocalciferol” in the fields “title,” “abstract,” “text,” and “MeSH (medical subject headings)” or “keywords,” without applying language or date restriction. The bibliographic references of the items retrieved by the original search were also reviewed for identifying other pertinent investigations. Only those studies using standardized criteria for diagnosing migraine (e.g., those of the Headache Classification Committee of the International Headache Society) [14] and reporting original information about vitamin D status in migraine patients were finally included.

## 3. Overview on Epidemiological Data 

**Figure 1:**
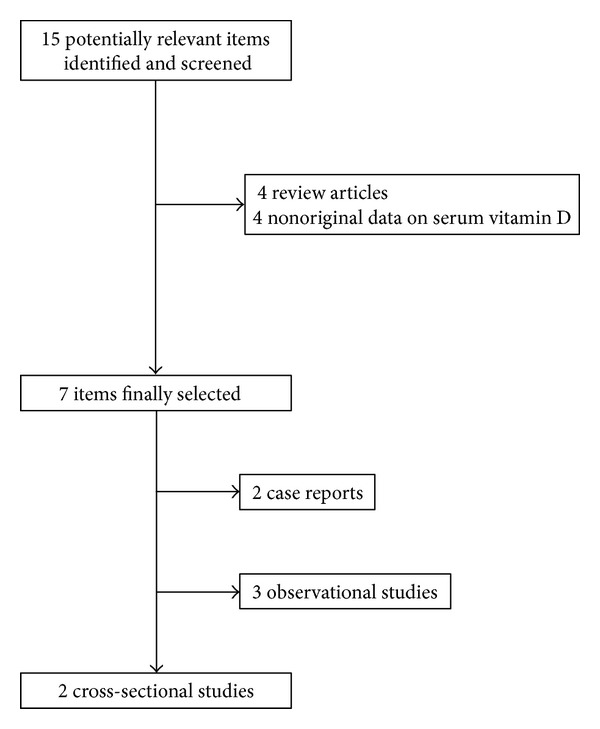
Flow diagram of study selection about the association between total serum vitamin D values and migraine.

The electronic search performed according to the previously defined criteria produced 15 items after exclusion of duplicates across the different scientific databases. Accurate reading of title, abstract, and full text (when available) allowed the exclusion of 8 items, which did not report original information about vitamin D status in patients with migraine (Figure 1). Seven studies were finally included in this systematic review, 3 observational, 2 cross-sectional, and 2 case reports (Table 1) [15–21].

Thys-Jacobs originally described the cases of two premenopausal women with migraine and low levels of total serum vitamin D (19.8 ng/mL and 15 ng/mL), who were treated with a combination of vitamin D and calcium for the presence of late luteal phase symptoms [15]. In both cases, the therapy with calcium and vitamin D was associated with substantial reduction of migraine attacks within 2 months of therapy. In a case report, the same author reported the cases of two postmenopausal women with migraine and low serum vitamin D levels (i.e., <5 ng/mL and 17 ng/mL), who were also treated with combination of vitamin D (1,200–1,600 IU daily) and calcium and displayed a considerable alleviation of both frequency and duration of migraine attacks [16]. These anecdotal reports paved the way to a subsequent series of studies, which directly investigated the potential relationship existing between vitamin D status and migraine.

Wheeler systematically reviewed records from 54 consecutive chronic migraine patients (48 women and 6 men; age range 17–73 years), who had serum vitamin D measured [17]. Vitamin D insufficiency (≤30 ng/mL) and deficiency (≤20 ng/mL) were observed in 40.7% and 14.8% of patients, respectively.

In a cross-sectional study, Kjaergaard et al. assessed vitamin D status in 248 nonsmoker migraine patients (184 females and 64 males; mean age 50 ± 11 years) and in 6121 nonsmoker control subjects without migraine (2820 females and 3301 males; mean age 60 ± 12 years) [18]. The concentration of total serum vitamin D was found to be marginally but significantly lower in cases than in controls (21.2 ± 6.5 versus 22.4 ± 7.2 ng/mL; *P* < 0.05). In the same study, the authors assessed total vitamin D status in 74 smoker migraineurs (54 females and 20 males; mean age 49 ± 10 years) and in 1432 nonsmoker control subjects without migraine (686 females and 746 males; mean age 57 ± 12 years) but failed to find any significant difference between cases and controls (28.1 ± 11.2 versus 27.6 ± 8.2 ng/mL; *P* = ns). Accordingly, in multivariable logistic regression analysis, no significant association was found between migraine and total serum vitamin D in both smokers (OR, 1.21; 95% CI, 0.57–2.55) and nonsmokers (OR, 1.03; 95% CI, 0.67–1.57).

Khorvash et al. investigated vitamin D status in 66 migraine patients (49 women and 17 men; mean age 36 ± 9 years) [19]. Vitamin D insufficiency (12–30 ng/mL) and deficiency (<12 ng/mL) were recorded in 66.7% and 13.6% of patients, respectively. In an investigation by the same team of authors, vitamin D status was assessed in 76 migraine patients (55 women and 21 men; mean age 33 ± 11 years) [20]. In agreement with previous data, vitamin D insufficiency (12–30 ng/mL) and deficiency (<12 ng/mL) were observed in 68.4% and 13.2% of patients, respectively.

Finally, Zandifar et al. performed a cross-sectional investigation including 105 patients with migraine (80 females and 25 males; mean age 33 ± 1 years) and 110 healthy controls (89 females and 21 males; mean age 32 ± 1 years) [21]. No significant difference was observed in the concentration of total serum vitamin D between cases and controls (13.6 ± 0.9 versus 13.2 ± 1.2 ng/mL; *P* = ns). The prevalence of vitamin D insufficiency (10–20 ng/mL) and deficiency (<10 ng/mL) was also similar between cases and controls (insufficiency: 34.3% versus 30.0%, *P* = ns; deficiency: 45.7% versus 51.8%; *P* = ns). Interestingly, no significant association was also observed between vitamin D concentration and frequency of migraine attacks, duration of migraine attacks, and positive family history of migraine.

## 4. Conclusions

Due to the high prevalence and the substantial clinical and economical burden placed on society, migraine should now be regarded as a public healthcare issue [11, 22]. The current approach for prevention of episodic migraine, as recently endorsed by the American Headache Society (AHS) and the American Academy of Neurology (AAN), is based on administration of topiramate, divalproex/sodium valproate, propranolol, and metoprolol [23]. Among nutritional supplements classified as “probably effective,” only riboflavin (i.e., vitamin B2) is included in the AHS/AAN guidelines. As regards the therapy of migraine, the AAN evidence-based guidelines currently advocate the use of specific agents (e.g., triptans or dihydroergotamine) in patients with moderate migraine and nonsteroidal anti-inflammatory drugs (NSAIDs) or aspirin plus acetaminophen in those with mild-to-moderate migraine attacks [24]. Even in this case, however, riboflavin was the only nutritional supplement for which an acceptable scientific support was acceptable.

Taken together, the current scientific evidence in support of a putative relationship between vitamin D deficiency and migraine is largely unsubstantial. The two separate case reports, including 4 women, showed a favourable effect of vitamin D supplementation on migraine severity, but these were obviously small studies and not placebo controlled. As regards the observational investigations, the frequency of vitamin D deficiency was found to be comprised between 13.2% and 14.8% of all migraine patients (Table 1). It is noteworthy that the burden of vitamin D deficiency in the general population ranges from 22% to 42% across different ages and genders [25, 26], and these figures are not really different from those reported in patients with migraine. The results of the two cross-sectional studies are even more controversial. In one of these, neither the concentration of total serum vitamin D nor the prevalence of vitamin D deficiency was found to be significantly different between controls and patients with migraine [21]. In the other study, a significant association between serum levels of vitamin D was found in nonsmokers but not in smokers [20]. It is noteworthy, however, that the significance of this modest association was completely lost after adjustment for age, body mass index, education level, alcohol consumption, and physical exercise, thus confirming that no apparent relationship exists between total serum vitamin D levels and migraine.

Among the studies that have been excluded from this systematic review due to the lack of data about serum vitamin D levels, one deserves special mention. Motaghi et al. assessed the relationship between two vitamin D receptor (VDR) gene polymorphisms (i.e., TaqI f/F and FokI t/T) and migraine in a cross-sectional study including 103 patients with episodic migraine (85 females and 18 males; mean age 34 ± 1 years) and 100 healthy nonmigraineurs (78 females and 22 males; mean age 35 ± 1 years) [27]. Interestingly, both heterozygote genotypes were significantly more prevalent in migraine patients than in controls (OR 1.81; 95% CI 1.03–3.18; *P* = 0.02 for TaqI and OR, 2.91; 95% CI, 1.47–5.77; *P* = 0.001 for FokI, resp.). Although these interesting findings do not provide definitive evidence for a causal association between VDR gene and migraine, the VDR has a broad expression in the central nervous system, where the binding to its agonists produces an important (pleiotropic) anti-inflammatory effect [28]. Since neurogenic inflammation is an essential part of migraine pathogenesis [12, 13], further studies are indeed advisable to establish whether the interplay between VDR agonists and VDR gene polymorphisms may have a role in preventing the development or worsening of migraine attacks.

The demand for vitamin D testing is dramatically increasing worldwide, often inappropriately [29]. The results of our analysis of clinical trials that have investigated the association between migraine and total serum vitamin D suggest that this relationship lacks reliable scientific support, so that this condition does not represent a valid indication for testing. Although larger cross-sectional or randomized prospective trials are obviously needed to draw definitive conclusions, vitamin D supplementation for prevention or treatment of migraine seems also unadvisable at this point in time. This is noteworthy, since the notion that vitamin D supplementation is absolutely safe has been recently challenged, especially in the elderly [30].

## Figures and Tables

**Table 1:** Synthesis of epidemiological studies exploring the association between vitamin D and migraine.

Author	Study design	Study population	Outcome	Reference
Thys-Jacobs, 1994	Case report	2 premenopausal women with migraine	Migraine attenuation after therapy with vitamin D	[15]
Thys-Jacobs, 1994	Case report	2 postmenopausal women with migraine	Migraine attenuation after therapy with vitamin D	[16]
Wheeler, 2008	Observational	54 migraine patients	Vitamin D insufficiency or deficiency observed in 40.7% and 14.8% of patients	[17]
Kjaergaard et al., 2012	Cross-sectional	248 nonsmoker migraine patients and 6121 controls; 74 smoker migraine patients and 1432 controls	Serum vitamin D marginally lower in cases than in controls in nonsmokers but not in smokers	[18]
Khorvash et al., 2013	Observational	66 migraine patients	Vitamin D insufficiency or deficiency observed in 66.7% and 13.6% of patients	[19]
Mottaghi et al., 2013	Observational	76 migraine patients	Vitamin D insufficiency or deficiency observed in 68.4% and 13.2% of patients	[20]
Zandifar et al., 2014	Cross-sectional	105 migraine patients and 110 controls	Serum vitamin D not significantly different between cases and controls	[21]
